# Best abstracts of the NVMO conference

**DOI:** 10.1007/s40037-013-0042-7

**Published:** 2013-02-08

## Introduction

During the yearly conference of the Netherlands Association of Medical Education, which took place in Maastricht on 15–16 November 2012, the awards for the best PhD thesis, scientific paper, regular paper and poster were given. The award-winning abstracts and the abstracts that were nominated are presented in this section of *Perspectives on Medical Education*. The best PhD thesis will be presented separately in one of the forthcoming issues of PME. The awards were been made available by the publisher of PME, Bohn Stafleu van Loghum (Springer Media).

## BEST SCIENTIFIC PAPER

### **Clarifying students’ feedback-seeking behaviour in clinical clerkships**

#### Bok GJ^1^, Teunissen PW^2^, Spruijt A^1^, Fokkema JPI^3^, van der Vleuten CPM^2^, van Beukelen P^1^, Jaarsma ADC^4^

##### ^1^Faculty of Animal Medicine, Utrecht University, ^2^Maastricht University, ^3^Sint Lucas Andreas Hospital, VU University Medical Centre, ^4^Academic Medical Centre—University of Amsterdam

###### **Context**:

 Why and how do students seek feedback on their performance in the clinical workplace and which factors influence this? These questions have remained largely unanswered in research into workplace learning during clinical clerkships. Research on feedback has focused mainly on feedback providers. Whether and how feedback recipients actively seek feedback are under-examined issues. Research in organizational psychology has proposed a mechanism whereby feedback seeking is influenced by motives and goal orientation mediated by the perceived costs and benefits of feedback. Building on a recently published model of residents’ feedback-seeking behaviour, we conducted a qualitative study to explore students’ feedback-seeking behaviours in the clinical workplace.

###### **Methods**:

 Between April and June 2011, we conducted semi-structured face-to-face interviews with veterinary medicine students in years 5 and 6 about their feedback-seeking behaviour during clinical clerkships. In the interviews, 14 students were asked about their goals and motives for seeking feedback, the characteristics of their feedback-seeking behaviour and factors influencing that behaviour. Using template analysis, we coded the interview transcripts and iteratively reduced and displayed the data until agreement on the final template was reached.

###### **Results**:

 The students described personal and interpersonal factors to explain their reasons for seeking feedback. The factors related to intentions and the characteristics of the feedback provider, and the relationship between the feedback seeker and provider. Motives relating to image and ego, particularly when students thought that feedback might have a positive effect on image and ego, influenced feedback-seeking behaviour and could induce specific behaviours related to students’ orientation towards particular sources of feedback, their orientation towards particular topics for and timing of feedback, and the frequency and method of feedback-seeking behaviour.

###### **Conclusions**:

 This study shows that during clinical clerkships, students actively seek feedback according to personal and interpersonal factors. Perceived costs and benefits influenced this active feedback-seeking behaviour. These results may contribute towards the optimizing and developing of meaningful educational opportunities during clerkships.

## BEST PAPER

### **Teaching, testing and training of professional behaviour: what is the benefit?**

#### Mak-van der Vossen M^1, 2^, Peerdeman S^1, 3^, Kleinveld A^1, 4^, Kusurkar R^1^

##### ^1^VUmc School of Medical Sciences, Institute for Education and Training, ^2^Department of General Medicine and Elderly Care Medicine, VU University Medical Centre, ^3^Department of Neurosurgery, VU University Medical Centre, ^4^Department of Clinical Genetics, VU University Medical Centre

###### **Background**:

 The VUmc School of Medical Sciences has developed an ‘Educational line on Professional Behaviour’, in which the teaching, testing and training of professional behaviour (PB) are described. In this programme different teachers judge students repeatedly during their medical study on their PB. If a student is judged to have ‘unsatisfactory’ PB, an individual guidance programme is started for him/her that involves the assessing teacher who has given the unsatisfactory judgement and the teacher of the subsequent course. To evaluate this approach towards remediation of unprofessional behaviour, the following questions were posed:

1. What happened to the students who received an ‘unsatisfactory’ judgement on PB?
2. What are students’ opinions about this approach?
3. What are teachers’ opinions about this approach?

###### **Methods/intervention**:

 A first ‘unsatisfactory’ judgement on PB leads the coordinator of PB to have a discussion with the student, preferably along with the assessing teacher. The student prepares a report of this discussion that is sent to the assessing teacher. The student is invited to discuss this report with the teacher of the subsequent course in order to enable guidance from this teacher in formulating the learning goals for the subsequent course. The last step is not obligatory. The student can compensate an unsatisfactory judgement by obtaining a satisfactory judgement in the subsequent course. The teacher of the subsequent course is trained to write qualitative feedback on the assessment form about the steps the student has taken to meet his learning goals.

If the student gets a second ‘unsatisfactory’ judgement on PB, the PB coordinator, with the permission of the Examination Committee of the institute, establishes contact with the teacher of the subsequent course. The subsequent teacher is informed in advance about the unsatisfactory PB of the student. The consent of the student for this is not considered to be necessary. This process is called ‘forward feeding’. Thus the student formulates his learning goals along with the PB coordinator and the subsequent teacher.

A report of the PB judgements of the students of all the 6 years of VUmc School of Medical Sciences, starting in the year 2010 (September) up to March 2012 was compiled. Students and teachers involved were asked for their opinions 6 months after the above-mentioned intervention was carried out.

###### **Results**:

 The total number of students in the academic year 2010–2011 was 2,533. Of these, 38 students received an unsatisfactory judgement on their PB in this year, for 6 of whom this was their second unsatisfactory judgement. These students were followed up to March 2012. In March 2012, out of the 38 unsatisfactory judgements in 2010, 23 students did not receive a subsequent unsatisfactory judgement and 9 received a second/third unsatisfactory judgement. Six of these 38 students had terminated their medical studies in March 2012, out of which 4 had two or more unsatisfactory judgements.

The feedback about the intervention from the students and the teachers was positive. The unsatisfactory judgements and the intervention created awareness among the students about their unsatisfactory PB, thus giving them an opportunity to improve in the subsequent course. The new approach helped the teachers to understand the impact of an unsatisfactory judgement assigned by them, and they felt ‘rewarded’ for their effort in teaching and assessing PB. A small group of students did not seem to benefit from the guidance, which lead to frustration among the teachers.

###### **Practical implications**:

 This approach of forward feeding and involvement of teachers in the remediation of PB enables early detection and effective remediation of unsatisfactory PB. Clear instructions and simple consultation options give teachers the confidence to give an unsatisfactory judgement in doubtful cases, allowing for marginal unprofessional behaviour to be detected. It is important to train teachers and inform the students adequately about the procedure for assessing PB.

## BEST POSTER

### **Is Facebook a suitable alternative for an electronic learning environment?**

#### Doets M, van den Broek WW

##### Erasmus MC Desiderius School, Erasmus University Medical Center

###### **Problem**:

 Because students increasingly use the social platform ‘Facebook’ for study-related discussions, ‘Blackboard’ (the official learning environment of Erasmus University medical faculty) is used less frequently. Also, as the number of posts on the Blackboard discussion boards has decreased, teaching staff have less insight into the issues that concern their students. To evaluate the possibilities of Facebook as an alternative to Blackboard, a pilot was carried out in the first Master year where Facebook was used as a communication tool for students and staff.

###### **Method**:

 A (closed) Facebook group was opened for all students participating in a specific week of the curriculum. Students were asked (in class and on Blackboard) to enrol in the Facebook group. Messages were posted on Facebook and students and staff took part in discussions. All lecture materials were still available via Blackboard. At the end of the week students were asked to fill out an online evaluation form.

###### **Results**:

 Of a possible 230 students, 123 enrolled in the Facebook group and two staff members actively participated in the discussions. During the specified week, 46 messages were posted on Facebook whereas earlier the same year 16 posts were made on Blackboard during 1 week. A total of 81 students completed the evaluation form, of which 67 were a member of the Facebook group (82 %); the remaining 14 students did not use Facebook. Facebook users more frequently looked on Facebook (76 % multiple times a day) than on Blackboard (63 % multiple times a day). They considered that Facebook made a positive contribution to the curriculum (54 %), e.g. because staff members are more involved and Blackboard is not up to date. Fifty percent of them considered Facebook to be more user-friendly than Blackboard.

Students not using Facebook considered that it made a negative contribution to the curriculum (65 %), e.g. because they found the use of Facebook unprofessional and because they do not like to mix private and educational matters.

###### **Conclusion and discussion**:

 Students are not unanimous about the use of Facebook as an educational tool; some students cannot or will not use Facebook. Because a medical school cannot oblige students to start using it, Facebook is not able to fully replace official channels. We will continue to stimulate the use of Blackboard so that no students or staff are excluded; however, the modernization of Blackboard is a priority for the coming year. Facebook can be used as a platform to involve students in the curriculum and to stimulate low-profile contacts between students and staff. Also, in 2012 the Erasmus MC Desiderius School started a Facebook ‘fan page’ with news and activities related to the medical curriculum.

## NOMINATED ABSTRACTS

### **Development of the serious game GeriatriX for interns in elderly care. Let’s play!**

#### Lagro J^1^, Veugelers M^2^, Huijbregts-Verheyden F^2^, van Litsenburg A^1^, Olde Rikkert MGM^1^

##### ^1^Department of Geriatric Medicine, Radboud University Nijmegen Medical Centre, ^2^Institute for (bio) Medical Education, Radboud University Nijmegen Medical Centre

###### **Introduction**:

 The current medical curriculum is disease-oriented and less focused on patient-oriented goals and preferences. Students should be better trained in multimorbidity and learn to question the standard diagnostic and therapeutic approach and weigh its appropriateness in the context of the individual patients with their specific preferences and multimorbidity.

###### **Methods**:

 To train students this complex medical decision-making in an attractive and safe way, we developed the serious game GeriatriX, as a multidisciplinary effort. In GeriatriX students weigh the following three criteria: (1) patient preferences, (2) appropriateness and (3) costs of medical care. We chose for a serious game because it is fun and fits in with an active, playful and adult learning method.

###### **Results**:

 GeriatriX was developed in a short period of 6 months (Fig. 1). It contains the same medical problem (anaemia) in three different elderly patients (context). This challenges students to explore different diagnostic and therapeutic strategies and gives insight into the consequences and costs of their choices. While playing they automatically receive feedback on their choices with respect to patient centredness, medical appropriateness and costs of medical care.

**Fig. 1:**
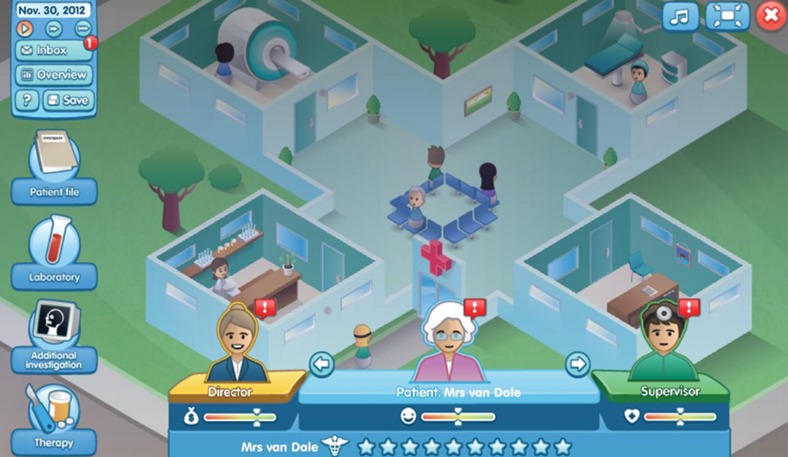
Serious game GeriatriX’s screen in which the supervisors for the three score domains are shown: The director for the cost-effectiveness; the supervisor for the medical appropriateness; the patient for the patient centredness. The scores are registered in the *bars below* them and in the overview *panel* at the *left*

###### **Conclusions**:

 GeriatriX is an innovative, challenging and promising educational tool. The next step to implement GeriatriX in the medical curriculum and research its merits.

### **Computer-supported collaborative learning in the medical workplace: students’ experiences on formative peer feedback of a Critical Appraisal of a Topic paper**

#### Koops WJM^1^, van der Vleuten CPM^2^, de Leng B^2^, Snoeckx L^2^

##### ^1^Máxima Medisch Centrum, ^2^Maastricht University

###### **Background**:

 Medical workplace learning consists largely of individual activities, since workplace settings do not lend themselves readily to group learning. An electronic Learning Management with System Computer-Supported Collaborative Learning (CSCL) could enable learners at different workplace locations to discuss personal clinical experiences at a distance to enhance learning.

###### **Aim**:

 To explore whether CSCL-enabled structured asynchronous discussions on an authentic task has additional value for learning in the medical workplace.

###### **Methods**:

 Between January 2008 and June 2010, we conducted an exploratory evaluation study among senior medical students who were engaged in clinical electives. Students wrote a Critical Appraisal of a Topic paper about a clinical problem they had encountered and discussed it in discipline homogeneous subgroups on an asynchronous forum in a CSCL environment. A mixed method design was used to explore students’ perceptions of the CSCL arrangement with respect to their preparation and participation, the design and knowledge gains. We analyzed the messages recorded during the discussions to investigate which types of interactions occurred.

###### **Results**:

 Students perceived knowledge improvement of their papers. The discussions were mostly task-focused. The students considered an instruction session and a manual necessary to prepare for CSCL. A high amount of sent messages and a high activity in discussion seem to influence scores on perceptions: ‘participation’ and ‘knowledge gain’ positively.

###### **Conclusion**:

 CSCL appears to offer a suitable environment for peers to provide formative feedback on a Critical Appraisal of a Topic paper during workplace learning. The CSCL environment enabled students to collaborate in asynchronous discussions, which positively influenced their learning.

### **Training supervisors in providing feedback: does it make sense?**

#### Zanting A^1^, Witkowska-Stabel M^2^, Stegers-Jager K^2^, Visser B^2^, Jousma F^2^, Visser L^3^

##### ^1^Ikazia Hospital Rotterdam, ^2^Erasmus University Medical Center, Rotterdam, ^3^St. Elisabeth Hospital, Tilburg

###### **Problem and research question**:

 In (postgraduate) medical education, there is an increasing emphasis on feedback as a learning tool (Norcini and Burch 2007). In order to improve the quality of feedback, clinical supervisors are trained in giving feedback, but the effects of training are unclear. In this study, we addressed the question whether a training in providing feedback improved the quality of written feedback given to interns. This study was conducted at the Department of Neurology of the St. Elisabeth Hospital Tilburg, the Netherlands.

###### **Methods**:

 At the request of the above department, a 5-h, on-site training on giving feedback was organized. Clinical supervisors of interns attended the training that consisted of theory and practice on oral and written feedback. The focus of the training was on giving feedback that (1) specifically describes an intern’s behaviour, (2) includes strong points as well as points to improve and (3) includes tips how to improve. Eight weeks after the training, participants reconvened with the facilitator to allow exchange of experiences and discussion of possible difficulties.

To measure the outcomes of the training, we restricted the study to written feedback given by clinical supervisors to interns of the Erasmus University Medical Center (MC), Rotterdam, the Netherlands. We used feedback forms that are integrated in the interns’ logbooks and are routinely filled out by their supervisors. We collected the forms filled out by the participants of the training in Tilburg who supervised neurology interns of the Erasmus MC. The ‘before’ assessment period started 3 months before and ended at the moment of the training. The ‘after’ assessment started after the second meeting and also lasted 3 months. For each period, 40 forms were collected. During the same periods, the same number of forms was collected among supervisors of neurology interns at the Erasmus MC in Rotterdam. These supervisors did not attend the training and acted as the control group. The forms were categorically analyzed on (1) specificity of feedback, (2) presence of positive as well as improvement points, and (3) presence of tips for improvement.

###### **Results**:

 In the intervention group, the amount of specific feedback on the forms increased from 59 % before to 95 % after the training (*χ*_(1)_^2^ = 14.6, *p* < 0.001). The feedback focussed in more cases on specific medical competencies (67.5 vs. 97.5 %, *χ*_(1)_^2^ = 12.5, *p* < 0.001). Supervisors also more often (55–75 %) mentioned points that should be improved, but this was not significant (*p* = 0.061). Finally, providing residents with tips how to improve, increased after training from 17.5 to 47.5 % (*χ*_(1)_^2^ = 8.2, *p* < 0.01). No such changes were observed in the control group.

###### **Discussion**:

 In this study, we showed that a short, practical training can be useful to improve the quality of written feedback. The training led to more specific feedback and more often included tips on how to improve. We studied the effects until 3 months after the training. Whether the effects continued to exist, should be subject of further research.

###### **Reference**:

 Norcini J, Burch V. Workplace-based assessment as an educational tool: AMEE Guide No. 31. Med Teach. 2007;29:855–71.

### **How do doctors collaborate according to one of their collaborative partners?**

#### van der Lee N^1^, Westerman M^1^, Fokkema JPI^1^, van der Vleuten CPM^2^, Scherpbier AJ^3^, Scheele F^4^

##### ^1^Department of Education, St. Lucas Andreas Hospital, Amsterdam, ^2^Department of Educational Development and Research, Faculty of Health, Medicine, and Life Sciences, Maastricht University, ^3^Institute for Medical Education, Faculty of Health, Medicine, and Life Sciences, Maastricht University, ^4^Department of Medical Education, VU University Medical Center, Amsterdam

###### **Introduction**:

 In their practice, doctors frequently collaborate with professionals from other disciplines. Research shows that this collaboration is not always successful and can negatively influence provided patient care. Within the setting of Dutch obstetrical healthcare, the collaboration between gynaecologists and midwives is seen as a causal factor in the relatively high perinatal morbidity rate, compared with other European countries. However, it is unknown what difficulties exist in this collaboration. We therefore explored the perspective of midwives regarding their collaboration with gynaecologists and their perspective on areas for improvement.

###### **Methods**:

 In a previous study, performed in 2010, we explored what gynaecologists could improve in their performance according to patients, midwives, nurses, general practitioners, and hospital boards. In this current study, we re-examined the qualitative data of midwives concerning gynaecologists’ collaborative performance to gain insight into areas for improvement. From the midwives’ dataset, specific remarks on collaborative performance were selected and analyzed using a template based on a validated model for interdisciplinary collaboration containing two domains and ten factors (1). The first, inter-relational domain (between team members), contains four factors; 1 ‘mutual acquaintanceship’, 2 ‘mutual trust’, 3 ‘shared goals and vision’, 4 ‘client-centred orientation versus other allegiances’. The second, inter-organizational domain (within an organization), beholds six factors; 5 ‘formalisation tools’ (e.g. protocols), 6 ‘information exchange’, 7 ‘connectivity’ (opportunities to meet and discuss with each other), 8 ‘support for innovation’, 9 ‘leadership’ en 10 ‘centrality’. New factors were added to the template if necessary.

###### **Results**:

 According to the midwives, gynaecologists’ performance can be improved in the inter-relational as well as in the inter-organizational domain. Within the inter-relational domain, midwives report that they would like gynaecologists to gain a better understanding on the midwives activities and responsibilities (factor 1). Within the inter-organizational domain, midwives express the need for more protocols on the responsibilities of each profession and on how obstetrical care should be provided (factor 5). There is also a need for more possibilities to meet up and discuss, for example, provided care and incidents (factor 7).

A newly found factor, ‘hierarchy’, was added to the template. According to midwives, gynaecologists tend to have a strongly hierarchical attitude towards midwives which should be diminished to facilitate collaboration.

###### **Conclusions**:

 According to midwives, current collaboration between gynaecologists and midwives in the Netherlands has to be improved on several factors inside and outside the inter-relational and organizational domains of interdisciplinary collaboration. Gaining insight into what gynaecologists can improve in their collaboration with midwives provides valuable information on the areas of collaboration between these professions that are to be improved. To improve the collaboration, research and interventions should focus on these areas.

###### **Reference**:

 D’Amour D, Goulet L, Labadie JF, Martin-Rodriguez LS, Pineault R. A model and typology of collaboration between professionals in healthcare organizations. BMC Health Serv Res. 2008;8:188.

### **How do physicians learn? The distribution of Kolb’s learning styles among physicians and their potential application in the design of continuing medical education (CME). A systematic review and meta-analysis**

#### Ten Thije JJH^1^, van Stiphout F^1^, Westers P^2^, ter Braak EWMT^1^

##### ^1^Department of Internal Medicine, University Medical Centre, Utrecht, ^2^Centre of Biostatistics, Julius Center for Health Sciences and Primary Care, Utrecht

###### **Introduction**:

 The rapidly growing body of medical knowledge together with time constrains of physicians call for efficient CME. According to the learning style hypothesis (LSH), application of learning styles may be used to increase educational efficiency. It is unknown whether physicians’ learning styles measured with the learning styles inventory (LSI) are typical for specific specialties. We therefore reviewed the literature and additionally investigated the evidence to apply the LSH.

###### **Methods**:

 We performed a systematic review and meta-analysis. We searched Medline, Embase, ERIC and PsychINFO, including studies that used Kolb’s LSI in physicians.

###### **Results**:

 Thirteen studies enrolling a total of 1,315 physicians of seven specialities were eligible. Subsequent analyses revealed statistically significant prevailing learning styles for: internal medicine and surgery (‘converging’), psychiatry, occupational medicine and general practice (‘assimilating’) and paediatrics (‘accommodating’) (goodness-of-fit test, all *p* < 0.02). Only 4/13 studies investigated application of Kolb’s LSI based on the LSH.

###### **Discussion**:

 It is possible to determine typical prevailing learning styles for the above specialities. However, little evidence supports the efficiency of application of learning styles for the design of CME.

###### **Conclusion**:

 Future research should aim to justify the use of LSH or alternative theories for potential application in CME.

### Evaluation by prediction instead of opinions: accurate, efficient and less susceptible for response bias*

#### Schönrock-Adema J^1^, Lubarsky S^2^, Chalk C^2^, Steinert ZY^2^, Cohen-Schotanus J^1^

##### ^1^University Medical Centre Groningen, ^2^McGill University, Montreal, Canada

###### **Introduction**:

 Traditional evaluation methods often suffer from too low response rates, which brings along the risk of response bias in the evaluation outcomes. Recently, a new evaluation method was tested, in which students were asked to estimate the opinions of their peers rather than provide their own opinions. The prediction method was found to require a significantly smaller number of respondents for stable outcomes, while the outcomes of both methods were comparable (Cohen-Schotanus et al. 2010). The rationale behind the prediction method is that prediction reduces the influence of personal variables on respondents’ perceptions, which implies that fewer respondents are needed for reliable outcomes. The aim of this study was to find empirical support for this rationale. The previous study was (partly) replicated in an international study. In addition, we investigated whether gender, estimated general level of achievement, expected test results, and satisfaction after having completed the exam caused bias in the evaluation outcomes. Our hypotheses were that prediction (1) requires fewer respondents for stable average outcomes, and (2) is less susceptible to personal bias due to gender, level of achievement and mood than the traditional approach to course evaluation.

###### **Methods**:

 We carried this international study out among 210 first-year undergraduate medical students at McGill University in Montreal (Canada) and 371 first-year and 385 third-year undergraduate medical students at the University of Groningen (the Netherlands). Participants were randomly assigned to one of two conditions and completed a 10-item course evaluation form on a 4-point scale. At each site, half of the participants gave their personal opinions as usual (*opinion condition*), the other half completed the course evaluation by predicting the outcomes, i.e. by estimating the percentages of peers that would choose each of the answering options. We determined how many respondents were required for stable outcomes using an iterative process. This process implied that we compared the average outcomes of sub-samples repeatedly to those of the entire group, with one person added to the sub-sample in each subsequent comparison. If differences between the average outcomes of the sub-sample and those of the entire sample were consistently less than 5 %, we considered the outcomes stable. Differences between the prediction and opinion methods in the numbers of respondents required for stable outcomes were analyzed using *t* tests. We used MANOVA to analyze differences in evaluation outcomes between the prediction and opinion methods. To investigate the method’s susceptibility for bias, we included four potentially biasing variables: *gender*, *estimated general level of achievement*, *expected test result*, and *satisfaction after having completed the exam*. We examined differences between males and females and between students scoring high and low on the latter three variables using MANOVA.

###### **Results**:

 The response rates ranged between 70 % (third-year students in Groningen) and 95 % (McGill). The prediction condition required on average 26–28 respondents for stable outcomes, while the opinion condition required on average 67–79 respondents. The *t* tests showed that prediction required significantly fewer respondents than the traditional method across all samples (*p* < 0.001), while the outcomes achieved were fairly similar. Estimated general level of achievement did not bias outcomes. Gender and satisfaction after having completed the exam only biased opinion outcomes (in 1 and 2 study samples respectively). In both conditions, expected test results caused bias in 1 study sample.

###### **Discussion/conclusion**:

 Our replication supported previous outcomes that prediction required significantly fewer respondents for stable outcomes than the traditional method of course evaluation and that both methods produced comparable outcomes. Our study added to previous research as we found support for our hypothesis that prediction responses are less susceptible to bias than traditional opinions. Our findings support prediction as an accurate and efficient method of course evaluation.

###### **Reference**:

 Cohen-Schotanus J, Schönrock-Adema J, Schmidt HG. Quality of courses evaluated by ‘predictions’ rather than opinions: fewer respondents needed for similar results. Med Teach. 2010;32:851–6.

*This study is accepted for publication in Medical Education: Schönrock-Adema J, Lubarsky S, Chalk C, Steinert Y, Cohen-Schotanus J. What Would My Classmates Say? An International Study of the ‘Prediction Method’ of Course Evaluation.

### **Evaluating a new profession in the Dutch healthcare system: physician assistants versus anaesthesiology residents in preoperative screening**

#### Hettinga AM^1^, Tromp Meesters RC^2^, Scheffer GJ^1^, van den Brink GTWJ^3^, Postma CT^1^

##### ^1^University Medical Centre St. Radboud, Nijmegen, ^2^Diakonessenhuis Utrecht, ^3^Hogeschool of Arnhem and Nijmegen

###### **Background**:

 In 2000 physician assistants were introduced in the Dutch healthcare system. One of the work settings where physician assistants are working is the preoperative outpatient clinic at the department of anaesthesiology. To study the clinical skills of these new professionals, an OSCE was developed to assess and compare the clinical skills of residents and physician assistants working in preoperative care.

###### **Summary of work**:

 A five-station OSCE was developed by three anaesthesiologists and two physicians with experience in OSCE development. Nine physician assistants, recently graduated or in the last part of their master, and 11 second year residents in anesthesiology participated in the OSCE. Participants were judged on history taking, physical examination, communication and their medical record, including risk assessment and preoperative risk reduction strategies. Cronbach’s *α* was calculated for the different aspects of assessment. Results were analyzed by *t* test.

###### **Summary of results**:

 The Cronbach’s *α* for the four aspects of assessment varied between 0.67 and 0.84. The results of this OSCE did not show significant differences between the clinical skills of physician assistants and residents.

###### **Conclusion**:

 Although the size of our sample was fairly small, this study provides a first view on the clinical skills of physician assistants engaged in preoperative care. Observing the outcomes of this study, physician assistants seem to perform at a similar level as residents, regarding preoperative care.

